# Preadmission Statin Prescription and Inpatient Myocardial Infarction in Geriatric Hip Fracture

**DOI:** 10.3390/jcm10112441

**Published:** 2021-05-31

**Authors:** Seth M. Tarrant, Raymond G. Kim, Jack M. McDonogh, Matthew Clapham, Kerrin Palazzi, John Attia, Zsolt J. Balogh

**Affiliations:** 1 Department of Traumatology, John Hunter Hospital, Lookout Rd, New Lambton Heights, NSW 2305, Australia; seth.tarrant@uon.edu.au (S.M.T.); raymond.kim@health.nsw.gov.au (R.G.K.); Jack.McDonogh@hnehealth.nsw.gov.au (J.M.M.); 2 School of Medicine and Public Health, University of Newcastle, Callaghan, NSW 2308, Australia; John.Attia@newcastle.edu.au; 3 Hunter Medical Research Institute, New Lambton Heights, NSW 2305, Australia; Matthew.Clapham@hmri.org.au (M.C.); Kerrin.Palazzi@hmri.org.au (K.P.)

**Keywords:** hip fracture, statins, pharmacoprotection, mortality, geriatrics

## Abstract

Statins have been shown to reduce myocardial infarction (MI) in cardiac and vascular surgery. MI is common in hip fracture. This study aims to investigate whether statins decrease MI in hip fracture surgery and reduce mortality resulting from MI. Patients aged 65 years and above with a low-energy hip fracture were identified between January 2015 and December 2017. Demographics, comorbidities, predictive scores, medications and outcomes were assessed retrospectively. The primary outcome was inpatient MI. The secondary outcome was inpatient mortality resulting from MI, for which fatal and non-fatal MI were modelled. Regression analysis was conducted with propensity score weighting. Hip fracture occurred in 1166 patients, of which 391 (34%) were actively taking statins. Thirty-one (2.7%) patients were clinically diagnosed with MI. They had a higher inpatient mortality than those who did not sustain an MI (35% vs. 5.3%, *p* < 0.0001). No reduction was seen between statin use and the occurrence of MI (OR = 0.97, 95% CI: 0.45–2.11; *p* = 0.942) including Fluvastatin-equivalent dosage (OR = 1.00, 95% CI: 0.96–1.03, *p* = 0.207). Statins were not associated with having a non-fatal MI (OR 1.47, 95% CI: 0.58-3.71; *p* = 0.416) or preventing fatal MI (OR = 0.40, 95% CI: 0.08–1.93; *p* = 0.255). Preadmission statin use and associations with clinically diagnosed inpatient MI or survival after inpatient MI were not able to be established.

## 1. Introduction

The total number of geriatric hip fractures is rising globally [1]. The increasing burden of geriatric fractures has major socio-economic consequences [2]. This population is highly vulnerable to morbidity and mortality, with cardiovascular disease common [3]. Clinically silent myocardial infarction has shown to occur in over one third of hip fracture patients during admission, with half occurring pre-operatively [4].

Optimising management of cardiac complications may lessen morbidity and prevent mortality. In hip fractures, statin use has been shown to be associated with a decrease in all-cause mortality [5,6]. The cause for this mortality reduction has not been clarified. Statins are used in the population to reduce cholesterol via inhibition of HMG CoA reductase but also have “pleiotropic effects”, including anti-inflammatory effects on coronary vasculature that promote plaque stability [7]. Systematic review and meta-analysis has demonstrated peri-operative statin treatment in statin-naïve patients reduces atrial fibrillation, myocardial infarction (MI) and duration of hospital stay in cardiac and non-cardiac surgery [8].

Patients with hip fractures have a high rate of cardiac co-morbidity and a high inpatient mortality [9], hence the reduction in MI rates and death from MI may be a critical component of statin-mediated reduction in all-cause mortality. We have conducted a retrospective cohort study aiming to investigate: (1) the proportion of patients who sustained a geriatric hip fracture who were prescribed statins on admission with Fluvastatin-equivalent weighting for dosage; (2) clinically diagnosed inpatient MI as per strict definition; (3) confounders of MI with subsequent statistical adjustment in analysis; (4) the rate of inpatient MI between patients prescribed and not prescribed statins; and (5) inpatient death resulting from MI. The hypothesis is that patients admitted on statins have reduced inpatient MI and resultant inpatient death.

## 2. Patients and Methods

### 2.1. Study Setting and Population

The study was conducted at a University-affiliated Level 1 Trauma centre located Australia. Patients were prospectively collected in the Australia New Zealand Hip Fracture Registry (ANZHFR) database from the start of 2015 by senior Orthopaedic clinical nurse consultants. The criteria for inclusion were sustaining a low-energy proximal femur fragility fracture and being aged 65 or over. Exclusion criteria included pathological fracture, peri-prosthetic and peri-implant fractures, high-energy mechanisms, polytrauma or patients that were transferred to a private facility for surgery. There were nil further inclusion/exclusion criteria applied, with all patients during the time frame studied. The study had ethical approval from the local Human Research Ethics Committee (AU201903-06) and is reported in accordance with the STROBE statement for retrospective cohort studies.

### 2.2. Data

Inpatient data from the index admission were collected between January 2015 and December 2017. Whilst functional, residential and mortality data are collected by the ANZHFR at the 30- and 120-day time points, they were not incorporated. Demographics, comorbidities and medications on admission, operative data and outcome details were collected for all patients. Statin dose and type was retrieved from the health record. “Antiplatelets” included aspirin, clopidogrel, aspirin and clopidogrel, dipyridamole and the antithrombotic combinations of aspirin and warfarin, clopidogrel and warfarin, and clopidogrel and direct-oral anticoagulant (DOAC). Age-adjusted Charlson Comorbidity Index (CCI) [10], American Society of Anesthiologists (ASA) score [11] and the Revised Cardiac Risk Index (RCRI; “Lee index”) [12] were calculated. The RCRI is a score with a minimum of 0 and a maximum of 6 and is composed of six criteria, for which 1 point is allocated per risk factor (Is the surgery high-risk? Does the patient have a history of ischaemic heart disease? Does the patient have a history of congestive cardiac failure? Does the patient have a history of stroke? Has the patient received pre-operative treatment with insulin? Is the patient’s pre-operative creatinine greater than 2 mg/dL?).

### 2.3. Outcomes

The primary outcome was MI during acute orthopaedic admission, assessed from chart review; the secondary outcome was inpatient mortality resulting from MI, derived from the ANZHFR during orthopaedic admission. To model the association between statin, MI, and death without introducing any bias (due to MI being on the hypothesised causal pathway between statin and death) a combined outcome measure was assessed: “Non-fatal MI” and “Fatal MI”. “Non-fatal MI” refers to a sustained MI in which the patient survived, and “Fatal MI” was an MI that clearly resulted in the death of a patient whilst admitted for the hip fracture; not an MI that was in conjunction with all-cause mortality.

Myocardial infarction (MI) was defined as per the European Society of Cardiology and the American College of Cardiology as: (a) pathological findings of an acute MI or (b) a typical rise and gradual fall in cardiac troponin concentrations combined with (i) ischaemic symptoms; (ii) pathological Q-waves in the ECG; (iii) ECG changes indicative of myocardial ischaemia (ST-segment elevation or depression); and/or (iv) coronary artery intervention [13]. Cardiac troponin (cTnI) was used in our institution (high-sensitivity TnI; Abbott, Abbott Park, IL, USA), with a concentration over 26 ng/L for males and 16 ng/L females considered to be above the 99th centile. All MI diagnosed were agreed on by either the consultant anaesthetist, physician or orthogeriatrician supported by troponin testing over the 99th centile. Patients that did not meet the definition (such as symptoms without elevated troponin levels or troponin elevations above the 99th centile without ECG changes) were excluded as MI. Routine “blanket” troponin testing is not performed in our institution for hip fractures.

With inpatient myocardial infarction rate approaching 40% in other studies looking into hip fracture [9], the fact that 30% [14] of the population is likely to be taking statins and that statins reduced the risk of MI by approximately 30% [15], it is estimated to be approximately 600 patients that will be needed for a power of 80% and setting *p* = 0.05.

### 2.4. Statistics

Demographic and disease-related information is presented as mean (standard deviation; SD) or median (quartiles 1 and 3; Q1, Q3), depending on distribution of continuous variables, or counts (%), and compared between patients who had an MI vs. those who had not, using independent t-test (continuous normally distributed), Mann–Whitney U test (continuous non-normal distribution), or Chi-square test (categorical).

The association between statin use (Y/N) and occurrence of MI was examined using logistic regression. Adjusted modelling using propensity score weighting was performed to account for potential confounding or selection bias. The odds of having statin use was modelled using logistic regression, and this was used to generate propensity scores; variables included age, sex, CCI, antithrombotics, beta blocker, ischaemic heart disease, ASA grade, RCRI, cerebrovascular disease, hypertension, previous acute pulmonary oedema, aortic aneurysm, chronic renal failure, diabetes mellitus, peripheral vascular disease, gastro-oesophageal reflux disease, chronic obstructive pulmonary disease, dementia, hypercholesterolaemia, degenerative skeletal disease, hypoparathyroidism, Parkinson’s disease and asthma (Supplementary Table S1). Stabilised inverse probability of group (SIPG) weights was then generated from the propensity scores and used in regression modelling. Balance between the statin use groups for propensity score weightings was confirmed graphically.

A dose–response association between statin medications and MI was examined using logistic regression. Continuous Fluvastatin-equivalent dose was the predictor [16]; if not on statin, dose was recorded as 0 mg.

Multinomial regression modelling was then used to examine the associations between statin use and severity of MI (3 level category as described above, with “No MI” as referent); odds ratios of non-fatal MI vs. no MI, and fatal MI vs. no MI are presented for statin use.

Regression model assumptions were checked for logistic regression; the linearity of independent variables and log odds was examined graphically, and the model fit was examined using the Hosmer–Lemeshow goodness of fit test. Odds ratios, with 95% confidence intervals (CI) and *p*-values are presented for logistic regression modelling. All statistical analyses were programmed using SAS v9.4 (SAS Institute Inc. Cary, NC, USA). Significance was set at α = 0.05 a priori.

## 3. Results

### 3.1. Demographic and Disease-Related Characteristics

Over the three-year period, 1166 patients were collected in the registry and were considered for analysis. Demographic and disease-related characteristics are listed (Table 1). Inpatient mortality occurred in 71 (6.1%) patients (statin: 21 (5.4%) vs. no statin: 50 (6.5%)) whilst 30-day mortality occurred in 124 (11%) patients (statin: 32 (8.2%) vs. no statin: 92 (12%)).

### 3.2. Propensity Score Model

The statin-user group were significantly younger (85 (8) vs. 82 (7); *p* < 0.001) and had a lower proportion of females (551 (71%) vs. 257 (66%); *p* = 0.061). These were adjusted for by propensity scoring (Supplementary Table S1). Revised cardiac risk index was not able to be fully adjusted for in the model (*p* < 0.001 to *p* = 0.016), but its individual components (IHD/CCF/CVD/DM/CRF) were matched.

For cardiac-related comorbidities there was a significantly higher proportion of patients with IHD, CVD, HTN, PVD, previous APO, aortic aneurysm, CRF, DM, GORD, dementia and hypercholesterolaemia in the statin group that were all adjusted for. For medications, statin users had higher prescription rates of antiplatelets, BB, CCB, ACE-I, ARB and spironolactone. These were able to be adjusted, except for CCB and ACE-I. Furthermore, there was a significantly increased proportion of statin users taking nitro vasodilators after propensity score matching, and a significantly decreased proportion taking corticosteroids (Supplementary Table S1).

### 3.3. Myocardial Infarction

Diagnosis of inpatient MI was made in 31 patients (2.7%), of which 30 underwent surgery. Three surgical patients (10%) sustained MIs pre-operatively. The first patient had a non-ST elevated MI (NSTEMI), with a Troponin I (TnI) of 222 ng/L on presentation, which increased to 350 on day 1 (D1), then was operated on D2 receiving a long intramedullary nail (IMN). The second patient had a NSTEMI with TnI 265 ng/L elevation on admission and cannulated screws on D4. The third had STEMI on admission and a TnI of over 9000 ng/L. A long IMN was performed on D4. All three patients survived to discharge and to 30 days. The one patient who did not receive surgery in the cohort had a pre-operative TnI of 500 ng/L, was 91 years of age, and did not recover post NSTEMI.

**Figure 1:**
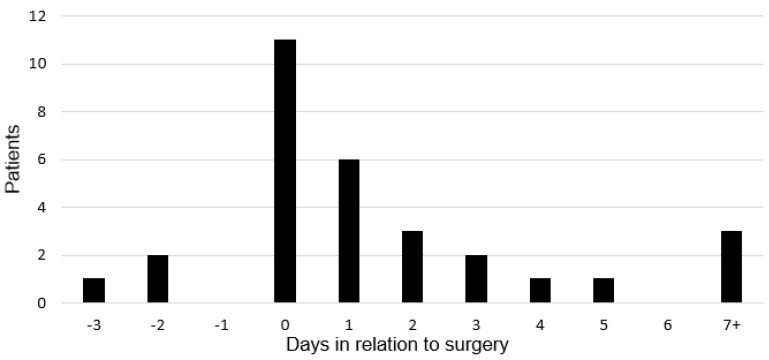
Timing of myocardial infarction in relation to surgery.

Eleven (37%) of 30 patients sustained MI within 24 h post-operation and six MIs (20%) occurred between 24 and 48 h post-operative (Figure 1). Of these 17 patients, seven died as inpatients; a mortality of 41% within this window. Three MIs (10%) occurred beyond D7 whilst still an inpatient for the hip fracture admission (D8, D11, D30). The patients who sustained MIs on D8 and D11 survived, however the patient diagnosed on D30 with TnI of over 17,000 ng/L died as an inpatient. MI was associated with increased inpatient mortality (MI vs. no MI: 35% vs. 5.3%; *p* < 0.0001).

### 3.4. Outcomes

#### 3.4.1. Primary Outcome: Association between Statin Use and Myocardial Infarction

Eighteen (2.3%) MIs occurred in statin-naïve patients, whilst 13 (3.3%) MIs occurred in statin users. No association between statin use and MI was seen in adjusted modelling (OR = 0.97, 95% CI: 0.45–2.11, *p* = 0.942). Continuous Fluvastatin-equivalent dose–response did not display an association (OR = 1.00, 95% CI: 0.96–1.03, *p* = 0.207).

#### 3.4.2. Secondary Outcome: Association between Statin Use, Myocardial Infarction, and Mortality

In the statin-naïve group, nine (1.2%) patients had a non-fatal MI, compared to 11 (2.8%) patients taking statins: a non-significant increased risk of non-fatal MI in statin-prescribed patients (OR = 1.47, 95% CI: 0.58–3.71; *p* = 0.416). Fatal MI occurred in nine (1.2%) statin-naïve patients compared to two (0.5%) patients taking statins, leading to non-significant lower odds of having a fatal MI when taking statins (OR = 0.40, 95% CI: 0.08–1.93; *p* = 0.255).

## 4. Discussion

This study investigates a cohort of geriatric hip fractures and the relation between statins, myocardial infarction during index admission and inpatient death. No reduction in the primary outcome of MI was seen in the group taking statins on admission. The secondary outcomes demonstrated that statins did not reduce inpatient mortality in patients who sustained an MI, or increase the odds of having a survivable, non-fatal MI.

### 4.1. Statin Use in Hip Fracture

On admission of this cohort, 34% were takings statins, which is at the higher end of the range for hip fractures. Global figures include 14% for Norway [5], 9.6% in Denmark [6], 17% for Japan [17] and 25% for Finland [18]. Australian orthopaedic trauma patients aged 60 have shown a similarly high statin usage of 31% [14]. Furthermore, 39% of our cohort were on antiplatelet agents similar to contemporary Danish (37%) [19] and Australian (40%) hip fracture cohorts [20].

In a prospective cohort of 364 Norwegian hip fractures, Juliebø et al. showed that pre-existing statin use was associated with a decrease in all-cause mortality (HR = 0.23, 95% CI: 0.08–0.68) over a median follow-up of 21 months. The authors attributed this mortality-reduction to pleiotropic effects reducing cardiovascular events and the inflammatory response related to injury and surgery [5], however cause of death and inpatient morbidity was not linked to this outcome. A recent 2020 Danish national database study of over 140,000 hip fractures by Jantzen et al. examined dose–response relationships of several medications against all-cause 30-day mortality. The 30-day mortality rate in statin users was 8.6%, whilst in statin-naïve patients it was 11%. For our study, we had almost identical 30-day mortality of 8.2% in statin group and 12% in the statin-naïve group. Jantzen et al. demonstrated a reduction in 30-day mortality for patients taking statins in both unadjusted (HR: 0.80, 95% CI: 0.75–0.85, *p* < 0.0001) and adjusted (HR: 0.87, 95% CI: *p* < 0.001) analysis (adjusted for age, sex, fracture type, CCI and drug dosage) [6]. Whilst being well powered, the authors caution that they cannot clarify the reasons for mortality reductions. Our study aimed to look at MI and resultant death as a potential reason for mortality reduction with the event rate lower than anticipated. Large national databases with a similar design as our study may be able to elucidate why statins are associated with decreased mortality.

### 4.2. Hip Fracture and MI

Our MI rate was 2.7% of the population with resultant inpatient mortality of 35%. Other Australian hip fracture studies have cited an inpatient MI rate of 3.8% based on clinical diagnosis [21]. In a large coding-based study of hip fracture morbidity from the USA, inpatient MI occurred at 1.9% in low volume hospitals, 2.1% in intermediate, and 2.2% in high volume hospitals [22]. A more recent US survey using the National Surgical Quality Improvement Program database showed a post-operative (within 30 days) MI rate of 1.8%, with patients operated on within 24 h having a 2.0% MI rate, 1.7% for 24–48 h and 1.6% after 48 h [23]. The incidence of post-operative MI in a Danish hip fracture population within 30 days was 1.15% (95% CI: 1.09%–1.22%) [24]. Our peak of MI for inpatients occurred close to surgery. The incidence of MI after hip fracture and cause of death is not well described post-discharge in the literature due to difficulties in diagnosis.

These figures based on databases and clinical diagnosis may underestimate the true prevalence of MI in hip fracture. Myocardial infarction has been cited at 36% in hip fractures when Troponin T (TnT) measurements are used as diagnostic criteria. More than half of all patients have ECG changes, with half of these elevations occurring pre-operatively and many patients displaying no clinical symptoms. In more than half of patients, the MI was the first manifestation of CAD [9]. In our study, a clinical diagnosis of MI was only made in 4 of 31 patients pre-operatively, suggesting there is a burden of cardiovascular events not being clinically detected within our institution.

### 4.3. Statin Use and MI in Hip Fracture

Admission on statins did not reduce the rate of inpatient MI in adjusted analysis, nor was there a dose-dependent relationship between statin and MI. The risk of sustaining a non-fatal MI or fatal MI was not significantly different for statin-naïve and statin-prescribed patients. Chopra et al. demonstrated in a systematic review and meta-analysis of RCTs that peri-operative statin treatment reduced atrial fibrillation, MI and duration of hospital stay, however 13 of the 15 RCTs were of vascular and cardiac surgery, and only one study, DECREASE-IV, examined orthopaedic surgery [8]. In this study, orthopaedic patients made up 16% of the trial participants and were not individually studied. The statin groups showed a trend toward reduced MI events and death, but did not reach significance. Preloading of statins was for a median of 34 days before surgery [25]. Despite hip fracture surgery being high risk, these RCTs in an elective setting may hold little relevance.

Three RCTs have been registered looking at the effects of statins in hip fracture. The Statin in Hip Fracture (STAFF) trial based in Brest, France, was designed to randomise rosuvastatin (5 or 20 mg) vs. placebo in 1200 patients, and to look at non-fatal VTE, acute coronary syndrome, non-fatal stroke, other acute ischemic arterial events, or all-cause death. The study was terminated by the steering committee, as recruitment was more difficult and slower than expected [26].

The Post-Operative Statin for Thromboprophylaxis & Cardiovascular Outcomes Protection (POST-OP) trial based in New York, aimed to conduct a 30-patient trial for statin-naïve patients over 65 with hip fracture, randomised to atorvastatin 40 mg vs. placebo, and aimed to recruit 1000 patients to examine anti-inflammatory effects and heart injury. This pilot study was published after only recruiting four hip fracture patients, with the feasibility of performing a large trial questioned due to: (i) ethical concerns of assigning patients with pre-morbid cardiac disease to placebo; (ii) pre-existing statin use in 40% of the population; and (iii) multiple co-morbidities that clashed with inclusion criteria [27].

The third RCT based in Melbourne, Australia, aims to recruit 200 patients and assign ivabradine (f-channel receptor blocker), 80 mg of atorvastatin, both, or neither. Patients will be assessed with ECG, cardiac echo, cardiac biomarkers and CT coronary angiography. The primary outcome will be post-operative TnI measurements to define myocardial injury [28], however this has not been reported seven years post publication of methodology.

### 4.4. Limitations

Several limitations to this study exist. There is a low event rate of clinically defined MI, and the wide confidence intervals highlight this. Based upon the MI rates in this study it is underpowered: a post-hoc power calculation (setting the alpha at 0.05 and power of 80%) would require 4263 patients in each arm (statin and non-statin groups) to detect a difference in inpatient MI rates. With a low clinical detection of MI, answering this question adequately would need either more patients or an increase in the event rate with prospective biochemical detection of cardiac events. Only detecting MI within the inpatient admission period and not over a longer follow-up, such as within 90 days, may also mean that MI that could be related to the hip fracture episode is missed.

We chose to follow up patients during index admission only, to control for diagnosis of MI and likely cause of death. Inpatient events, however, are dictated by length of stay, which is variable and often dependent on extrinsic factors beyond clinical control. Our institutional mean acute length of stay is reported at 10 days [29], however this is less for patients who can return to high-level long-term care facilities and are usually more frail. Of note is the fact that patients taking statins were significantly younger than the statin-naïve population, however residence was not adjusted for.

Another limitation is the inability for the propensity score to account for all independent variables. RCRI still showed a significant difference between the two groups. All other cardiac and non-cardiac comorbidities were adjusted. Not all medications were adjusted for, with CCB, ACE-I, nitrovasodilators and corticosteroids showing significant differences after propensity weighting. In Jantzen et al.’s large database study both ACE-Is and CCBs failed to be associated with a 30-day mortality despite large numbers, so adjustment for these drugs is unlikely to be critical [6]. The effect of nitrovasodilators and corticosteroids on outcome is not known in the hip fracture literature to our investigation.

The focus of the failed POST-OP RCT [27] and unreported RCT of Rudd et al. [28] is the anti-inflammatory effects of statins, proposed to help increase long-term survival [5]. Hip fracture is associated with a heightened inflammatory response which represents an independent predictor for mortality and morbidity [30], however, the long-term anti-inflammatory effects of the statins in an immune-primed and cardiovascularly-vulnerable hip fracture cohort was not able to be examined by this study.

### 4.5. Strengths

This study was performed at a single, high-volume institution where surgical and medical practice is conducted with a shared-care acute orthogeriatric model. Diagnosis of MI was based upon clinical investigation and not reliant on coding from multiple institutions. We demonstrated that patients taking statins have far greater cardiac-related comorbidity which needs to be appropriately adjusted for in future observational studies, as it seems likely that prospective studies will be difficult to power from clinically based MI. The modelling used in this study had comprehensive adjustment for comorbidity confounders. Whilst Jantzen et al. adjusted for CCI only, we have adjusted for comorbidities not incorporated in the CCI, of both cardiac and non-cardiac nature [6]. Death from MI was lower in patients taking statins, and despite being underpowered, this remains a promising avenue for a database with capabilities to appropriately adjust for confounders. We have also demonstrated that the majority of inpatient MI occurs in the peri-operative period before institutional mean discharge.

## 5. Conclusions

This study has examined a cohort of geriatric hip fracture of which approximately one third of patients were taking statins. Inpatient myocardial infarction (MI) occurred in 2.7% of hip fracture patients. The association with preadmission statin use and the reduction in inpatient MI and subsequent death from inpatient MI were not able to be adequately established.

## Figures and Tables

**Table 1:** Demographic and disease-related characteristics, by statin use.

	No Statin	Statin	Total	
Characteristic	(*n* = 775)	(*n* = 391)	(*n* = 1166)	*p*-Value
Age (years; mean, SD)	85 (8)	82 (7)	84 (8)	<0.001
Sex (Female; *n*, %)	551 (71%)	257 (66%)	808 (69%)	0.061
Type (*n*, %)				
Atorvastatin		184 (47%)		
Simvastatin		91 (23%)		
Rosuvastatin		87 (22%)		
Pravastatin		27 (6.9%)		
Surgery Performed (yes; *n*, %)	758 (98%)	382 (98%)	1140 (98%)	0.906
CCI (mean, SD)	6 (2)	7 (2)	6 (2)	0.357
ASA grade (mean, SD)	3.1 (0.7)	3.1 (0.6)	3.1 (0.7)	0.169
RCRI (median, Q1–Q3)	0 (0,1)	1 (0,2)	1 (0,1)	<0.001
Cardiac-related Comorbidity				
Ischaemic Heart Disease	160 (21%)	157 (40%)	317 (27%)	<0.001
Congestive Cardiac Failure	115 (15%)	59 (15%)	174 (15%)	0.910
Cerebrovascular Disease	216 (28%)	155 (40%)	371 (32%)	<0.001
Hypertension	451 (58%)	280 (72%)	731 (63%)	<0.001
Peripheral Vascular Disease	45 (5.8%)	35 (9.0%)	80 (6.9%)	0.045
Previous Acute Pulmonary Oedema	37 (4.8%)	41 (10%)	78 (6.7%)	<0.001
Existing Atrial Fibrillation	215 (19%)	5 (16%)	220 (19%)	0.503
Valvular heart disease	25 (3.2%)	14 (3.6%)	39 (3.3%)	0.750
Prosthetic valve	6 (0.8%)	7 (1.8%)	13 (1.1%)	0.119
Aortic aneurysm	18 (2.3%)	19 (4.9%)	37 (3.2%)	0.020
Pacemaker	23 (3.0%)	15 (3.8%)	38 (3.3%)	0.430
Non-cardiac Comorbidity				
Chronic Renal Failure	81 (10%)	69 (18%)	150 (13%)	<0.001
Diabetes Mellitus	104 (13%)	116 (30%)	220 (19%)	<0.001
Gastro-oesophageal Reflux Disease	215 (28%)	134 (34%)	349 (30%)	0.022
Chronic Obstructive Pulmonary Disease	117 (15%)	62 (16%)	79 (6.8%)	0.734
Malignancy	153 (19%)	71 (18%)	224 (19%)	0.517
Dementia	235 (30%)	82 (21%)	317 (27%)	<0.001
Hypercholesterolaemia	67 (8.6%)	210 (54%)	277 (24%)	<0.001
Medications				
Antiplatelet	235 (30%)	223 (57%)	458 (39%)	<0.001
Beta blocker	188 (24%)	169 (43%)	357 (31%)	<0.001
Calcium channel blocker	100 (13%)	86 (22%)	186 (16%)	<0.001
Angiotensin converting enzyme inhibitor	91 (12%)	122 (31%)	269 (23%)	<0.001
Angiotensin Receptor Blocker	91 (12%)	79 (20%)	170 (15%)	<0.001
Frusemide	195 (25%)	111 (28%)	306 (26%)	0.237
Spironolactone	50 (6.5%)	39 (10%)	89 (7.6%)	0.032
Ezetimibe	7 (0.9%)	6 (1.5%)	13 (1.1%)	0.332
Nitro vasodilators	124 (16%)	73 (19%)	197 (17%)	0.251
NSAID	23 (3.0%)	9 (2.3%)	32 (2.7%)	0.511
Corticosteroids	59 (7.6%)	18 (4.6%)	77 (6.6%)	0.051

ASA, American Society of Anesthesiologists; CCI, Charlson Comorbidity Index; NSAID, non-steroidal anti-inflammatory drug; RCRI, Revised Cardiac Risk Index.

## Data Availability

The data presented in this study are available on request from the corresponding author. The data are not publicly available due to IRB policy. Institutional Review.

## Supplementary Materials

The following are available online at https://www.mdpi.com/article/10.3390/jcm10112441/s1, Table S1: Demographic and disease-related characteristics adjusted with Propensity Weighting.
