# Supplementary material for: Preadmission Statin Prescription and Inpatient Myocardial Infarction in Geriatric Hip Fracture
Source: J Clin Med. 2021 May 31;10(11):2441. doi: 10.3390/jcm10112441 (PMC8199133; doi:10.3390/jcm10112441)
Supplement: Supplementary file 1 [file jcm-10-02441-s001.zip › jcm-1218061-supplementary.pdf]

**Supplement Table S1.** Demographic and disease-related characteristics adjusted with Propensity Weighting.

| Characteristic | Crude | | *p*-Value | Weighted | | *p*-Value |
|---|---|---|---|---|---|---|
| | No Statin | Statin | | No Statin | Statin | |
| Age (yrs; mean, SD) | 85 (8) | 82 (7) | <0.001 | 84 (8) | 84 (7) | 0.521 |
| Sex (Female; *n*, %) | 551 (71%) | 257 (66%) | 0.061 | 528 (70%) | 232 (67%) | 0.352 |
| Type (*n*, %) | | | | | | |
| Atorvastatin | | 184 (74%) | | | 143 (43%) | |
| Simvastatin | | 91 (23%) | | | 101 (30%) | |
| Rosuvastatin | | 87 (22%) | | | 68 (20%) | |
| Pravastatin | | 27 (6.9%) | | | 22 (6.6%) | |
| Surgery Performed (yes; *n*, %) | 758 (98%) | 382 (98%) | 0.906 | 753 (99%) | 382 (98%) | 0.457 |
| CCI (mean, SD) | 6 (2) | 7 (2) | 0.357 | 7 (3) | 7 (2) | 0.210 |
| ASA grade (mean, SD) | | | 0.169 | | | 0.362 |
| 1 | 7 (0.9%) | 0 (0%) | | 6 (0.7%) | 0 (0%) | |
| 2 | 110 (14%) | 64 (17%) | | 102 (14%) | 43 (12%) | |
| 3 | 439 (58%) | 229 (60%) | | 436 (58%) | 198 (57%) | |
| 4 | 203 (27%) | 88 (27%) | | 212 (28%) | 103 (30%) | |
| 5 | 2 (0.3%) | 2 (0.5%) | | 2 (0.2%) | 2 (0.7%) | |
| RCRI (median, range) | | | <0.001 | | | 0.016 |
| 0 | 438 (57%) | 132 (34%) | | 364 (48%) | 140 (40%) | |
| 1 | 216 (28%) | 144 (37%) | | 228 (30%) | 128 (37%) | |
| 2 | 93 (12%) | 70 (18%) | | 101 (13%) | 53 (15%) | |
| 3 | 21 (2.7%) | 36 (9.2%) | | 37 (4.8%) | 23 (6.5%) | |
| 4 | 6 (0.8%) | 8 (2.0%) | | 28 (3.7%) | 4 (1.1%) | |
| 5 | 1 (0.1%) | 1 (0.3%) | | 1 (0.1%) | 1 (0.1%) | |
| Cardiac-related Comorbidity | | | | | | |
| Ischaemic Heart Disease | 160 (21%) | 157 (40%) | <0.001 | 217 (29%) | 117 (34%) | 0.077 |
| Congestive Cardiac Failure | 115 (15%) | 59 (15%) | 0.910 | 142 (19%) | 54 (16%) | 0.194 |
| Cerebrovascular Disease | 216 (28%) | 155 (40%) | <0.001 | 243 (32%) | 113 (33%) | 0.857 |
| Hypertension | 451 (58%) | 280 (72%) | <0.001 | 472 (62%) | 230 (66%) | 0.204 |
| Peripheral Vascular Disease | 45 (5.8%) | 35 (9.0%) | 0.045 | 43 (5.6%) | 25 (7.3%) | 0.302 |
| Previous Acute Pulmonary Oedema | 37 (4.8%) | 41 (10%) | <0.001 | 51 (6.7%) | 25 (7.3%) | 0.730 |
| Existing Atrial Fibrillation | 215 (19%) | 5 (16%) | 0.503 | 156 (21%) | 63 (18%) | 0.329 |
| Valvular heart disease | 25 (3.2%) | 14 (3.6%) | 0.750 | 25 (3.3%) | 10 (3.0%) | 0.772 |
| Prosthetic valve | 6 (0.8%) | 7 (1.8%) | 0.119 | 5 (0.7%) | 5 (1.4%) | 0.284 |
| Aortic aneurysm | 18 (2.3%) | 19 (4.9%) | 0.020 | 21 (2.7%) | 12 (3.4%) | 0.571 |
| Pacemaker | 23 (3.0%) | 15 (3.8%) | 0.430 | 21 (2.7%) | 17 (5.0%) | 0.056 |
| Non-cardiac Comorbidity | | | | | | |
| Chronic Renal Failure | 81 (10%) | 69 (18%) | <0.001 | 114 (15%) | 50 (14%) | 0.752 |
| Diabetes Mellitus | 104 (13%) | 116 (30%) | <0.001 | 158 (21%) | 74 (21%) | 0.839 |

| | | | | | | |
|---|---|---|---|---|---|---|
| Gastro-oesophageal Reflux Disease | 215 (28%) | 134 (34%) | 0.022 | 214 (28%) | 109 (32%) | 0.253 |
| Chronic Obstructive Pulmonary Disease | 117 (15%) | 62 (16%) | 0.734 | 111 (15%) | 53 (15%) | 0.761 |
| Malignancy | 153 (19%) | 71 (18%) | 0.517 | 137 (18%) | 77 (22%) | 0.109 |
| Dementia | 235 (30%) | 82 (21%) | <0.001 | 225 (30%) | 105 (30%) | 0.854 |
| Hypercholesterolaemia | 67 (8.6%) | 210 (54%) | <0.001 | 182 (24%) | 92 (27%) | 0.368 |
| Medications | | | | | | |
| Antitplatelets | 235 (30%) | 223 (57%) | <0.001 | 303 (40%) | 158 (45%) | 0.090 |
| Beta blocker | 188 (24%) | 169 (43%) | <0.001 | 230 (30%) | 123 (35%) | 0.097 |
| Calcium channel blocker | 100 (13%) | 86 (22%) | <0.001 | 104 (14%) | 75 (22%) | <0.001 |
| Angiotensin converting enzyme inhibitor | 91 (12%) | 122 (31%) | <0.001 | 170 (22%) | 108 (31%) | <0.001 |
| Angiotensin Receptor Blocker | 91 (12%) | 79 (20%) | <0.001 | 102 (13%) | 56 (16%) | 0.240 |
| Frusemide | 195 (25%) | 111 (28%) | 0.237 | 220 (29%) | 111 (28%) | 0.237 |
| Spironolactone | 50 (6.5%) | 39 (10%) | 0.032 | 65 (8.6%) | 23 (6.6%) | 0.255 |
| Ezetimibe | 7 (0.9%) | 6 (1.5%) | 0.332 | 14 (1.9%) | 3 (0.9%) | 0.195 |
| Nitroavasodilators | 124 (16%) | 73 (19%) | 0.251 | 117 (15%) | 80 (23%) | <0.001 |
| NSAID | 23 (3.0%) | 9 (2.3%) | 0.511 | 26 (3.5%) | 14 (3.9%) | 0.722 |
| Corticosteroids | 59 (7.6%) | 18 (4.6%) | 0.051 | 61 (8.0%) | 10 (3.0%) | <0.001 |

ASA- American Society of Anesthesiologists, CCI- Charlson Comorbidity Index, NSAID- non-steroidal anti-inflammatory drug, RCRI- Revised Cardiac Risk Index.
